# Metabolic Responses of Carotenoid and Cordycepin Biosynthetic Pathways in *Cordyceps militaris* under Light-Programming Exposure through Genome-Wide Transcriptional Analysis

**DOI:** 10.3390/biology9090242

**Published:** 2020-08-21

**Authors:** Roypim Thananusak, Kobkul Laoteng, Nachon Raethong, Yu Zhang, Wanwipa Vongsangnak

**Affiliations:** 1 Interdisciplinary Graduate Program in Bioscience, Faculty of Science, Kasetsart University, Bangkok 10900, Thailand; roypim.th@ku.th; 2 Industrial Bioprocess Technology Research Team, Functional Ingredients and Food Innovation Research Group, National Center for Genetic Engineering and Biotechnology (BIOTEC), National Science and Technology Development Agency (NSTDA), Pathum Thani 12120, Thailand; 3 Department of Zoology, Faculty of Science, Kasetsart University, Bangkok 10900, Thailand; nachonase@hotmail.com; 4 Key Laboratory for Animal Genetics, Breeding, Reproduction and Molecular Design of Jiangsu Province, Yangzhou University, Yangzhou 225009, China; yuzhang@yzu.edu.cn; 5 Omics Center for Agriculture, Bioresources, Food, and Health, Kasetsart University (OmiKU), Bangkok 10900, Thailand

**Keywords:** *Cordyceps militaris*, carotenoid, cordycepin, light response, transcriptome analysis, genome-scale metabolic network

## Abstract

*Cordyceps militaris* is currently exploited for commercial production of specialty products as its biomass constituents are enriched in bioactive compounds, such as cordycepin. The rational process development is important for economically feasible production of high quality bioproducts. Light is an abiotic factor affecting the cultivation process of this entomopathogenic fungus, particularly in its carotenoid formation. To uncover the cell response to light exposure, this study aimed to systematically investigate the metabolic responses of *C. militaris* strain TBRC6039 using integrative genome-wide transcriptome and genome-scale metabolic network (GSMN)-driven analysis. The genome-wide transcriptome analysis showed 8747 expressed genes in the glucose and sucrose cultures grown under light-programming and dark conditions. Of them, 689 differentially expressed genes were significant in response to the light-programming exposure. Through integration with the GSMN-driven analysis using the improved network (*i*RT1467), the reporter metabolites, e.g., adenosine-5′-monophosphate (AMP) and 2-oxoglutarate, were identified when cultivated under the carotenoid-producing condition controlled by light-programming exposure, linking to up-regulations of the metabolic genes involved in glyoxalase system, as well as cordycepin and carotenoid biosynthesis. These results indicated that *C. militaris* had a metabolic control in acclimatization to light exposure through transcriptional co-regulation, which supported the cell growth and cordycepin production in addition to the accumulation of carotenoid as a photo-protective bio-pigment. This study provides a perspective in manipulating the metabolic fluxes towards the target metabolites through either genetic or physiological approaches.

## 1. Introduction

*Cordyceps militaris* is a well-known edible and medicinal fungus belonging to entomopathogenic fungi, which parasitizes a larvae or pupae of lepidopteran insects [1]. Since *C. militaris* produces a variety of bioactive compounds with functional properties, such as cordycepin (3′-deoxyadenosine), carotenoid, ergosterol and polysaccharide, it has long been used as dietary supplement and medicinal herb in East Asia, Southeast Asia and North America [2,3,4]. In addition to the commercial use of whole-cell mass or fruiting body, the bioprocess development through fermentation and downstream processes permits a prospect in the production of specific bioactive compounds as functional ingredients for diversified applications. Among these compounds, the cordycepin has been mostly mentioned in *C. militaris*, and its potent pharmaceutical function has been investigated for clinical trials against cancer [5]. Additionally, the carotenoid has attracted particular interest in its strong antioxidant activities [6].

In the fungal cultivation process, carotenoid is highly accumulated in the fruiting body of *C. militaris* culture with prolonged exposure to light. Recent studies revealed that the stimulation of carotenoid biosynthesis in *C. militaris* by light was attributed at the transcriptional level, where the up-regulations of the terpenoid synthase gene (*Cmtns*) [7] and flavohemoprotein gene (*Cmfhp*) [8] were detected. Although the carotenoid does not play a direct physiological role in the formation of fruiting body of *C. militaris*, it may act as a free radical scavenger to decrease the genotoxicity of nitrogen oxide and reactive oxygen species (ROS) under light exposure [9,10,11]. In addition to the bio-pigment, the contents of bioactive compounds in *C. militaris*, particularly cordycepin, were altered in response to the light condition [3,12,13]. A previous study of albinism in *C. militaris* grown under the light condition showed that cordycepin content in the albino strain was higher than that of the wild type [14]. However, the metabolic regulation governing the biosynthesis of these two key metabolites, carotenoid and cordycepin, remains unexplored. To date, a genome-scale metabolic model has enabled the optimization of *C. militaris* cultivation to enhance growth and cordycepin production by rational design of culture medium composition [15]. Moreover, there are earlier studies documenting the carbon source utilization in *C. militaris*, in which glucose and sucrose were often used as preferred carbon sources for cell growth, cordycepin and carotenoid production [16,17]. To gain more insight into the key metabolites, it is worth addressing the metabolic network of biosynthetic pathways of cordycepin, and other metabolites in *C. militaris* (e.g., carotenoid), which would accelerate the progress in this fungal field in aspects of fundamental knowledge and biotechnological production of value-added metabolites.

In this work, we therefore aimed to investigate the metabolic responses of *C. militaris* to light exposure by using integrative genome-wide transcriptome and genome-scale metabolic network (GSMN)-driven analysis. To obtain informative data, carbon source and light/dark exposure were subjected as variables for the fungal cultivation. The comparative transcriptomic analysis of the glucose and sucrose cultures grown under the light-programming and dark conditions was implemented for identifying the differentially expressed genes (DEGs). The genome-scale metabolic network of *C. militaris* was also improved for integrating with DEGs to analyze the key metabolites associated with the carotenoid and cordycepin biosynthetic pathways. This study postulates the metabolic response of *C. militaris* to the light-programming condition by cooperating in several pathways through the transcriptional expression of a set of metabolic genes, particularly in carotenoid and cordycepin biosynthesis, that offers a perspective in manipulating the pathways of targets through either genetic or physiological approaches.

## 2. Materials and Methods

### 2.1. Fungal Strain and Cultivation

**Figure 1:**
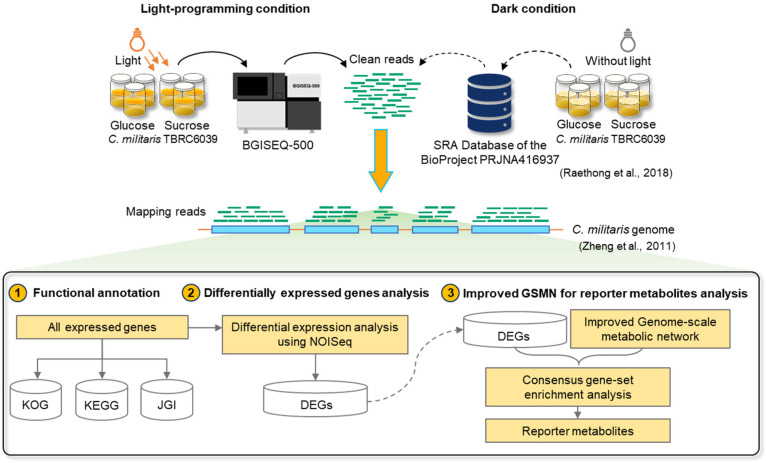
Systematic workflow of transcriptome analysis and genome-scale metabolic network-driven analysis of *C. militaris* strain TBRC6039.

*C. militaris* strain TBRC6039 was used in this study. Fungal cultures were grown in defined media, containing glucose or sucrose as carbon source, at static condition, 22 ± 2 °C, as detailed in the previous study [17]. For the carotenoid-producing condition, the fungal cultivation was conducted under the light-programming condition by switching the light (~1000 lux) and dark conditions at an interval of 12 h. All batch experiments were performed in three biological replicates. The samples of mycelial grown cultures were subjected to systematic analysis, as shown in Figure 1. For biomass measurement, the samples were collected at different time points of the cultivation, and filtered to separate the fungal cells from the fermented broth. Then, the fungal cells were dried using freeze dryer at −90 °C overnight. Dried mycelia were weighted for calculation of dry cell weight (DCW) and biomass productivity (g DCW L^−1^ h^−1^). For statistical data analysis, one-way analysis of variance (ANOVA) followed by post hoc Tukey’s test was used for multiple comparison. All statistical analyses were performed using the analytic tools in Microsoft Excel and Minitab (version 17) under significant difference (*p*-value ≤ 0.05).

### 2.2. Determination of Cordycepin and Carotenoid Contents of C. militaris Cultures

Fermented broths from the fungal cultures were filtered prior to high-performance liquid chromatography (HPLC) analysis. For the extracellular cordycepin measurement, the filtrated culture broth was subjected to the HPLC analysis using a HiQSil C18HS column (300 mm × 4.6 mm, 5 µM) at 40 °C, and a UV detector at a wavelength of 260 nm, in which 15% (*v*/*v*) methanol solution was used as a mobile phase at a flow rate of 1.0 mL/ min. To determine carotenoid content, dried mycelia were ground and subjected to the extraction of carotenoid using the acid-heating method [6]. The carotenoid content was then measured by the colorimetric method using a spectrophotometer with a wavelength of 445 nm.

### 2.3. RNA Sequencing, Reads Mapping, and Functional Annotation

Using a single carbon source (glucose or sucrose), the *C. militaris* cultures grown at the mid-logarithmic phase under the light-programming condition were harvested for RNA sequencing. Total RNA was extracted using the RNeasy mini kit (Qiagen). Equal amounts of total RNA samples extracted from three independent cultures, using each sugar as a sole carbon source, were taken and pooled together for RNA sequencing according to the other previous studies [17,18,19,20]. RNA quality and concentration were measured by the Agilent 2100 bioanalyzer. NanoDrop^TM^ was used to identify the purity of RNA samples. After that, the cDNA library construction and RNA sequencing were performed. To the end, paired-end reads were cleaned by removing adapters, and reads containing unknown bases (N) >5%, and the low-quality reads. RNA-Seq data or clean reads of the glucose and sucrose cultures of *C. militaris* grown under the light-programming condition were kept in FASTQ format files for depositing in the National Center for Biotechnology Information (NCBI) Sequence Read Archieve (SRA) under the BioProject accession number PRJNA579732.

To further identify the active metabolic trait involved in the carotenoid production of *C. militaris*, the earlier transcriptome data of the strain TBRC6039, which was grown in the same culture medium and temperature at the dark condition [17], were retrieved from the BioProject accession numbers of PRJNA416937 (BioSample: SAMN07969453 and BioSample: SAMN07969454). The clean reads of the four different cultures with two variable sets (glucose/sucrose and light-programming/dark conditions) were mapped back to the *C. militaris* strain CM01 genome [21] for read mapping, as illustrated in Figure 1, using the alignment package Burrows-Wheeler Aligner (BWA) [22]. SAMtools was used for estimating mapped reads to gene reference [23]. The genes that had a number of mapped reads higher than 100 were subjected to calculation of FPKM (fragment per kilobase of transcript per million mapped reads), which is a normalized estimation value of gene expression based on RNA-Seq data [24], in which the expressed genes were indicated by FPKM value ≥ 1. For annotation, EuKaryotic Orthologous Groups (KOG), Kyoto Encyclopedia of Genes and Genomes (KEGG) Orthology (KO) databases [25], and Joint Genome Institute (JGI) via MycoCosm portal [26] were used to perform functional assignment of the expressed genes.

### 2.4. Differentially Expressed Genes Analysis

To generate DEGs, the expressed genes of the fungal cultures grown in glucose or sucrose, at both the light-programming and dark conditions, were compared based on the distribution method by NOISeq-sim. Basically, NOISeq-sim can be used to identify DEGs from the pooled RNA-Seq experiments. NOISeq-sim therefore simulates technical replicates with default parameters, i.e., a percentage of the total number of reads (pnr) of 0.2, a number of simulated samples (nss) of 5, and a variability in sample total reads used to simulate samples (v) of 0.02. To estimate the DEGs by NOISeq-sim, the probability threshold (q) > 0.8 and |log_2_ fold change| > 1 were set [27,28]. The visualization of DEGs pattern was performed using Z-score transformation, which provided a way of standardizing FPKM data across glucose or sucrose cultures under the light-programming and dark conditions. It independently permitted the comparison of gene expression data. To illustrate, a heat map diagram was generated by using the heatmap.2 function in the R gplots package [29].

### 2.5. Improvement of the Genome-Scale Metabolic Network of C. militaris for Reporter Metabolites Analysis

To uncover the reporter metabolites, the previous genome-scale metabolic network (GSMN) of *C. militaris* (*i*NR1329) [15] was improved by introducing the genes and their associated metabolites and biochemical reactions involved in carotenoid biosynthetic pathway and sugar transport system, as previously reported [7,14,30]. The additional genes identified from the first GSMN of *C. militaris* (*i*WV1170) [31] together with extensive data mining from different databases were newly added into *i*NR1329, thereby enhancing the gene content of the improved GSMN in this study. Then, the KEGG annotation and the improved GSMN of *C. militaris* as scaffolds were integrated with the list of DEGs obtained from the glucose cultures grown under the light-programming and dark conditions for identification of the key pathways and metabolites using the consensus gene-set enrichment analysis and reporter metabolites analysis, respectively. Notably, the metabolite, which had a distinct up-directional *p*-value < 0.01, was identified as a reporter metabolite [32,33].

## 3. Results and Discussion

### 3.1. Growth Characteristics and Production of Carotenoid and Cordycepin of C. militaris on Different Carbon Sources

The growth characteristics and production of carotenoid and cordycepin of *C. militaris* TBRC6039 on different carbon sources are shown in Table 1. Among the two carbon sources, sucrose was preferable for *C. militaris* growth at the light-programming condition, similar to the previous study of fungal cultivation at the dark condition [17]. At the light-programming condition, the maximum specific growth rate (μ_max_) of 0.0122 ± 0.0017 h^−1^ was obtained in the sucrose culture, which was higher than that of the glucose culture (μ_max_ of 0.0116 ± 0.0040 h^−1^). However, the extracellular cordycepin titers of the cultures grown at the dark and light-programming conditions were not significantly different. A similar trend was observed in the both glucose and sucrose cultures. Light-programming conditions resulted in high growth and production of carotenoid contents in both glucose and sucrose cultures, which were significantly different from the dark condition, as shown in Table 1 and Supplementary File 1. The carotenoid contents were not dependent on the sugars tested, which might be presumably referred to the significant attribute of light exposure on the photo-protective pigment [34] rather than the carbon sources.

### 3.2. Genome-Wide Transcriptome of C. militaris

**Figure 2:**
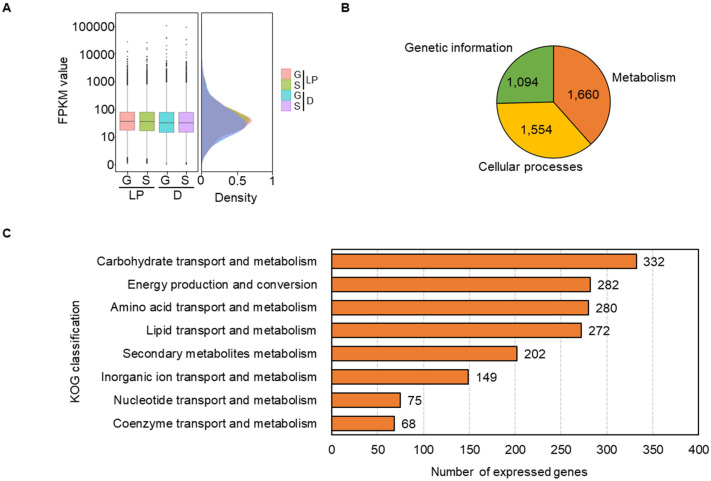
Assessed transcriptome data of *C. militaris* cells grown at different cultivation conditions. (**A**) Box plot and histogram of the FPKM distribution of the glucose (G) and sucrose (S) cultures grown at the light-programming (LP) and dark (D) conditions. (**B**) Functional categories of *C. militaris* TBRC6039 transcriptome using the KOG database. (**C**) Metabolic functional categories of expressed genes based on the KOG database.

To identify the active metabolic traits of *C. militaris* at the light-programming condition, the extracted RNA pools of the glucose or sucrose cultures of *C. militaris* TBRC6039 were sequenced and analyzed, as shown in Figure 1. The published transcriptome data of the *C. militaris* TBRC6039, which was grown in the same culture media and temperature at the dark condition [17], deposited in the BioProject accession numbers of PRJNA416937 (BioSample: SAMN07969453 and BioSample: SAMN07969454), was used for comparative analysis. As summarized in Table 2, a total of clean reads of 48.81 Mb and 48.79 Mb obtained from this study and a total of clean reads of 45.15 Mb and 44.87 Mb taken from previous work were then mapped to the *C. militaris* genome [21], resulting in the average total mapped reads of 82.57 ± 2.02%. Considering the removal of redundant expressed genes identified from overall conditions, 8747 expressed genes having the similar FPKM distribution were gained. The results are shown in Figure 2A and Supplementary File 2.

Based on KOG annotation, 4308 out of 8747 expressed genes were categorized into three main functional groups, including metabolism (1660 genes), cellular process (1554 genes) and genetic information (1094 genes). The results are illustrated in Figure 2B. Observably, the majority of expressed genes of *C. militaris* were associated with metabolic functions (see Figure 2C), which were involved in the carbohydrate transport and metabolism (332 genes), energy production and conversion (282 genes), amino acid transport and metabolism (280 genes), lipid transport and metabolism (272 genes), secondary metabolites metabolism (202 genes), inorganic ion transport and metabolism (149 genes), nucleotide transport and metabolism (75 genes), and coenzyme transport and metabolism (68 genes). Consistent with the KEGG annotation, the metabolism was mostly related to functional categories (Supplementary File 3). This result indicates that the metabolism of *C. militaris* was highly active in response to light and dark conditions.

### 3.3. Differentially Expressed Genes of C. militaris Cultures at Light-Programming and Dark Conditions

**Figure 3:**
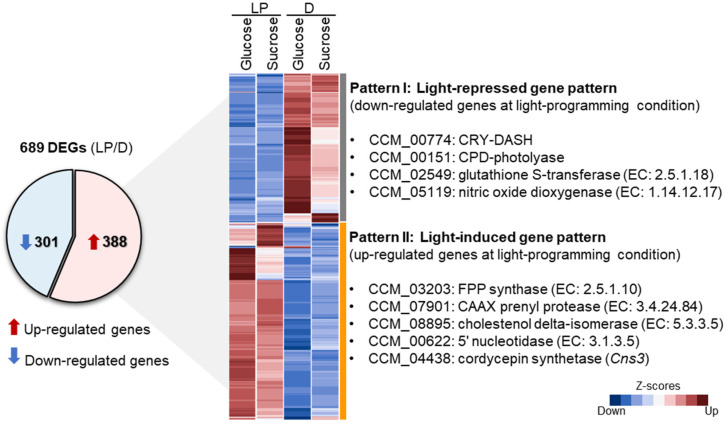
Differentially expressed genes (DEGs) in the glucose or sucrose cultures under light-programming (LP) and dark (D) conditions. A pie chart shows the number of DEGs. A heat map diagram shows different gene patterns. Each gene is colored by the standardized expression value using Z-score transformation across all different conditions.

To explore the transcriptional responses in the metabolic genes, a pairwise comparison of the expressed genes between light-programming and dark conditions of the glucose or sucrose cultures was performed. With the statistical criteria of NOISeq-sim (see Materials and Methods), 689 DEGs were significantly identified (q > 0.8 and |log_2_ fold change| > 1). The results are shown in Figure 3 and Supplementary File 4. Of the 689 DEGs, 388 were observed to be up-regulated and 301 were down-regulated. As illustrated in Figure 3, two main different patterns were identified, which included light-repressed gene pattern (Pattern I) and light-induced gene pattern (Pattern II), according to the gene sets with down- and up-regulated expressions at light-programming condition, respectively. Considering the Pattern I, a total of 301 genes of the glucose and sucrose cultures displayed low expressions at light-programming condition, whereas highly expressed genes were observed in both cultures grown at dark condition. Particularly, the CCM_00774 and CCM_00151 encoding for CRY-DASH and CPD-photolyase, respectively, which were identified as the photoreceptor genes in the *C. militaris* genome [35], were down-regulated in light-programming condition. On the other hand, some studies have reported that these genes, i.e., CRY-DASH and CPD-photolyase, were activated under light-stress condition [14,36]. So far, it has been known that CRY-DASH and CPD-photolyase are UV-damaged DNA repair enzymes, which utilize light energy to lyse UV-induced bonds between adjacent pyrimidine bases in DNA fragments, called photo-reactivation [37]. Possibly, the light-programing condition with light/dark cycle used for the cultivation in this study might not substantially induce the UV-damaged mechanism of the fungal cells, which acclimatized to growing under such cultivation conditions [38]. It has been further reported that the inhibition of the CRY-DASH resulted in high accumulation of carotenoid and cordycepin contents in *C. militaris* [35]. In our work, the down-regulation of CRY-DASH and CPD-photolyase genes might be cooperated with the up-regulation of the genes involved in carotenoid biosynthesis, such as CCM_03203 gene encoding for farnesyl diphosphate (FPP) synthase (EC: 2.5.1.10), which eventually enhanced carotenoid biosynthesis in *C. militaris* under the cultivation at light-programming condition. According to the previous study by Lou et al. [7], the CCM_03203 gene involved in light-induced carotenoid formation in *C. militaris* was suggested based on the transcriptome analysis.

In addition to the targeted metabolites (e.g., carotenoid), the particular enzymes (e.g., glutathione peroxidase) also act as free radical scavengers [39]. In this work, we found the down-regulated genes to be associated with a defense mechanism, such as CCM_02549 gene encoding for glutathione S-transferase (EC: 2.5.1.18) and CCM_05119 gene encoding for nitric oxide dioxygenase (NOD) (EC: 1.14.12.17) at light-programming condition. These enzymes display an important role in antioxidant activities to attenuate or prevent the cell damage by removing reactive species or free radicals (e.g., reactive oxygen species (ROS) and reactive nitric oxide species (RNS)) [40,41], which are known as signal molecules of defense responsive mechanisms of the cells. However, the previous report demonstrated that the NOD-coding gene (*Cmfhp*) was significantly up-regulated when *C. militaris* CM01 was treated with light [8], and the deletion of this gene resulted in enhancing nitric oxide (NO) content and lowering carotenoid content as compared with the wild type. Possibly, the down-regulation of the NOD gene found in our study might indicate the presence of a compensatory mechanism through the function of photo-protective pigment [42] to cope with the stress response or, in turn, that the light-programming condition did not largely induce the light stress, and the cell acclimatization occurred in contrast to the effect of the light exposure, as previously studied [8].

**Figure 4:**
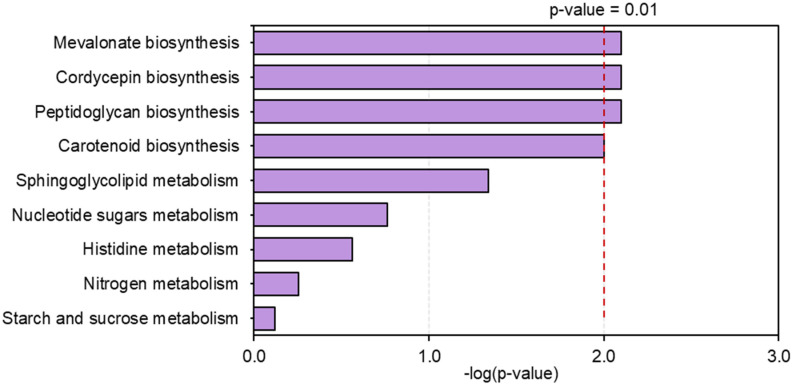
The functional enrichment analysis of differentially expressed genes using KEGG annotation.

Considering the Pattern II, a total of 388 genes in the glucose or sucrose cultures were highly expressed at light-programming condition, whereas low expressions of these genes were observed in both cultures grown at dark condition (Figure 3). As expected, our results found that the gene encoding for FPP synthase was significantly up-regulated. This enzyme catalyzes the conversion of geranyl diphosphate and isopentenyl diphosphate to farnesyl diphosphate, a common precursor for synthesizing carotenoid. In addition, the genes involved in steroid synthesis, such as the CCM_07901 gene encoding for CAAX prenyl protease (EC: 3.4.24.84), and the CCM_08895 gene encoding for cholestenol delta-isomerase (EC: 5.3.3.5), were also significantly up-regulated. After the functional enrichment analysis of DEGs using KEGG annotation, promisingly, the expressed genes involved in the mevalonate and carotenoid biosynthesis were enriched at light-programming condition (Figure 4 and Supplementary File 3), such as the genes encoding for beta-carotene 15,15′-dioxygenase (EC: 1.13.11.63), geranylgeranyl diphosphate synthase (EC: 2.5.1.29), mevalonate kinase (EC: 2.7.1.36), mevalonate diphosphate decarboxylase (EC: 4.1.1.33), and squalene synthetase (EC: 2.5.1.21). These enzymes are responsible for carotenoid biosynthesis via the mevalonate pathway [7,9,14]. These results supported the hypothesis that the up-regulation of the genes involved in carotenoid biosynthesis led to the high accumulation of carotenoid content in the fungal cells grown at light-programming condition. They might also indicate that *C. militaris* produced large amounts of carotenoid as a defense mechanism to light and oxidative stress. Very interestingly, the CCM_00622 gene encoding for 5′ nucleotidase (EC: 3.1.3.5) and the cordycepin-producing gene (*Cns3*: CCM_04438) encoding for cordycepin synthetase were significantly up-regulated in the carotenoid-producing cultures grown at light-programming condition. According to a previous study by Zhao et al. [43], the orthologous CCM_04438 gene involved in cordycepin biosynthesis in *Cordyceps kyushuensis* was also suggested to be a response to light exposure. These findings suggest that the fungal cells sustained the cordycepin biosynthesis at the light-programming condition through transcriptional regulation. Notably, as observed in the Pattern II, some genes were a response to a single carbon source under light-programming conditions. For example, the CCM_01308, CCM_04978, CCM_08838, and CCM_06082 genes were significantly up-regulated under sucrose as a carbon source and light exposure (Supplementary File 4). 

### 3.4. Metabolic Responses of C. militaris in Carotenoid and Cordycepin Biosynthetic Pathways to Light-Programming Condition

Emphasizing the biosynthesis of important metabolites (i.e., carotenoid and cordycepin), the GSMN of *C. militaris* was systemically improved by introducing the genes and their associated metabolites as well as biochemical reactions involved in the carotenoid biosynthetic pathway into the previous study reported by Raethong et al. [15]. Compared to the earlier network (*i*NR1329) [15], a total of 138 additional genes were identified from the GSMN of *C. militaris* (*i*WV1170) [31] together with extensive data mining from different databases. These included the genes related to the light-induced carotenoid formation from the previous studies [7,8]. Notably, the gene encoding for 2′, 3′-cyclic-nucleotide 2′-phosphodiesterase (EC: 3.1.4.16) previously identified by Wongsa et al. [44] was also introduced for expanding the metabolic routes towards the formation of cordycepin. The improved GSMN of *C. militaris*, called *i*RT1467, was further used for integrative analysis of transcriptome data. Eventually, *i*RT1467 contained a total of 1467 genes, 1184 metabolites and 1835 biochemical reactions (Supplementary File 5).

To identify the metabolic responses of *C. militaris* to the light-programming condition, the integrative analysis of transcriptome data was done using the *i*RT1467 as a scaffold. Interestingly, we found that the genes involved in carotenoid and cordycepin biosynthetic pathways were mainly up-regulated at the light-programming condition, such as CCM_03203 encoding for FPP synthase (EC: 2.5.1.10), CCM_03203 encoding for dimethylallyltranstransferase (EC: 2.5.1.1), cordycepin-producing gene (*Cns3*: CCM_04438) encoding for cordycepin synthetase, and CCM_00622 encoding for 5′-nucleotidase (EC: 3.1.3.5) with log_2_ fold change of 1.16, 1.16, 2.14, and 1.39, respectively. In addition to such metabolic pathways, the genes involved in the glyoxalase system were highly up-regulated at the light-programming condition. The glyoxalase system is responsible for detoxifying the methylglyoxal (MGO), which is generated by lipid peroxidation, glycation, and degradation of glycolytic intermediates [45]. Moreover, it has been reported that MGO is another type of the stress-associated signaling compounds as reactive carbonyl species (RCS), which are mainly produced by ROS-mediated lipid peroxidation [46,47]. Indeed, it is possible that the *C. militaris* cells might acclimatize to such stress by eliminating the toxic ROS through the glyoxalase system. Moreover, it could be explained that the phenotypic characteristics of *C. militaris* with high carotenoid content were a cellular response to overcome the light stress by up-regulation in transcriptional expression of the genes involved in the biosynthesis of carotenoid, which is known to be a potent bio-pigment with a photo-protective function against UV and oxidative damages.

The up-regulated expression of the genes in the cordycepin biosynthetic pathway found in the cultures grown at light-programming condition might explain the capability of the carotenoid-rich cells in sustaining the cordycepin production at the observed cordycepin titer (Table 1). In addition, the genes, CCM_08193 and CCM_03659, encoding for amine oxidase (EC: 1.4.3.4) were up-regulated at such conditions with log_2_ fold change of 1.87 and 1.24, respectively. This enzyme converts aminoacetone (AA) to MGO, and generates hydrogen peroxide (H_2_O_2_) and ammonia (NH_3_). To cope with MGO toxicity induced by the light stress, the enzymes involved in the glyoxalase system might be active for conversion of MGO to pyruvate, as indicated by the highly up-regulated expressions of the CCM_05147 gene encoding for methylglyoxal reductase (EC: 1.1.1.283) with log_2_ fold change of 12.38, and CCM_04150 and CCM_04156 genes coding for hydroxyacylglutathione hydrolase (EC: 3.1.2.6) with log_2_ fold change of 10.57 and 10.56, respectively. 

**Figure 5:**
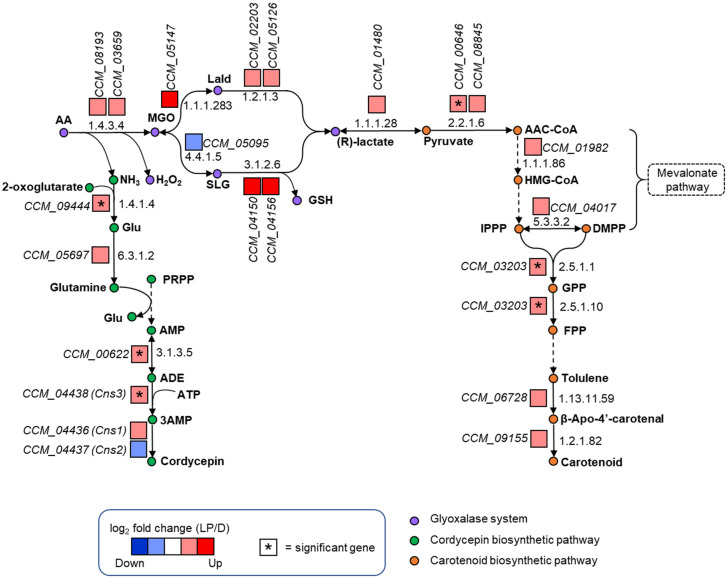
Metabolic responses of *C. militaris* to the light-programming condition through cordycepin and carotenoid biosynthetic pathways. Abbreviations: AA, aminoacetone; MGO, methylglyoxal; NH_3_, ammonia; H_2_O_2_, hydrogen peroxide; Lald, lactaldehyde; SLG, S-lactoylglutathione; GSH, glutathione; Glu, Glutamate; PRPP, 5-phospho-alpha-ribose 1-diphosphate; AMP, adenosine-5′-monophosphate; ADE, adenine; ATP, adenosine-5′-triphosphate; 3AMP, adenosine-3′-monophosphate; AAC-CoA, acetoacetyl-CoA; HMG-CoA, 3-hydroxy-3-methylglutaryl-CoA; IPPP, isopentenyl diphosphate; DMPP, dimethylallyl diphosphate; GPP, geranyl diphosphate; FPP, farnesyl diphosphate.

Focusing on ammonia, which is a metabolite product derived from the catalytic conversion of aminoacetone to MGO by amine oxidase, we found that the gene coding for ammonia transporter (CCM_06254) was significantly down-regulated in the carotenoid-producing culture induced by the light-programming condition. This might lead to the accumulation of ammonia in the fungal cells, and the responsive mechanism might be active to reduce the toxicity of the excess ammonia by converting the ammonia to cordycepin through transcriptional regulation. Alternatively, it is possible that ammonia was turned to glutamate, which is a precursor for cordycepin biosynthesis as a result of up-regulated gene CCM_09444 coding for glutamate dehydrogenase (EC: 1.4.1.4) with log_2_ fold change of 1.98. These results indicate that the fungal cell has a regulatory mechanism in cell growth and stress response for maintaining cell homeostasis at light-programming condition by co-expression of a set of genes involved in the glyoxalase system, cordycepin and carotenoid biosynthesis, as illustrated in Figure 5. This finding is consistent with the earlier reports on the light-responsive carotenoid and cordycepin biosynthesis [36]. 

To gain more understanding of how the cordycepin and carotenoid biosynthesis contribute to the light-responsive mechanism in *C. militaris*, the reporter metabolites analysis (see Materials and Methods) was then performed. As a result, it is clearly seen that the up-regulated genes found in light-programming condition were enriched in the particular reporter metabolites, such as adenosine-5′-monophosphate (AMP), adenosine-5′-diphosphate (ADP), adenosine-5′-triphosphate (ATP), uridine 5′-phosphate (UDP), glucose, UDP-glucose, thioredoxin dithiol, and 2-oxoglutarate, as listed in Table 3. Regarding on the reporter metabolites identified, we suggest that there was a correlation between the pools of metabolic energy and nucleotide, which were generated for supplying cell growth, cordycepin and carotenoid production under light-programming cultivation. These results indicate the metabolic responses of cordycepin and carotenoid biosynthesis in *C. militaris* at the particular condition, as depicted in Figure 5.

## 4. Conclusions

The integrative approach of genome-wide transcriptome and GSMN-driven analysis enables us to capture the metabolic responses of *C. militaris* to light/dark stimulus through transcriptional regulation, particularly in carotenoid and cordycepin biosynthesis. Notably, even though these studies were based on three biological replicates from *C. militaris* cultivation under light/dark conditions, low standard deviations and the desired power levels from statistical analysis were obtained, thus the data derived from such limited sample sizes were then subjected to transcriptome analysis with the integration of the GSMN-driven analysis using the improved network (*i*RT1467), showing good agreement in responsive genes and reporter metabolites during light exposure of *C. militaris*. At the light-programming condition, the up-regulation of a set of genes involved in several metabolic pathways was found, including the glyoxalase system, as well as carotenoid and cordycepin biosynthesis. These findings indicate a plausible mechanism in the acclimatization of this entomopathogenic fungus to the light/dark stress through a regulatory mode, at least at the transcription level, leading to enhanced mycelial growth with the altered phenotypes of pigment and some reporter metabolites. The sustainment of cordycepin production by the fungal cell under the light-programming condition also provides benefit in terms of the biotechnological production of biologically active metabolites in addition to the biomass production.

## Figures and Tables

**Table 1:** Growth and production of carotenoid and cordycepin of the glucose and sucrose cultures of *C. militaris* at light-programming and dark conditions.

Phenotypic Characteristics	Light-Programming		Dark	
	Glucose	Sucrose	Glucose	Sucrose
Maximum specific growth rate, µ_max_ (h^−^^1^)	0.0116 ± 0.0040	0.0122 ± 0.0017	0.0079 ± 0.0008 ^1^	0.0104 ± 0.0021 ^1^
Biomass productivity (g DCW L^−^^1^ h^−^^1^)	0.0122 ± 0.0018	0.0112 ± 0.0016	0.0096 ± 0.0013 ^1^	0.0088 ± 0.0004 ^1^
Extracellular cordycepin titer (mg L^−^^1^)	112.12 ± 0.32	95.65 ± 8.56	109.14 ± 11.54 ^1^	119.32 ± 14.06 ^1^
Carotenoid content (mg g DCW^−^^1^)	1.4692 ± 0.0122 ^a^	1.4590 ± 0.0052 ^a^	0.0244 ± 0.0051 ^b,2^	0.0234 ± 0.0095 ^b,2^

Note: Values are mean ± SD (n = 3). ^1^ Data were taken from the previous report [17]. ^2^ Analysis was conducted in this work by using the mycelial samples cultivated at the same condition [17]. ^a,b^ Different superscript letters in rows indicate statistically significant difference (*p*-value ≤ 0.05) by one-way ANOVA followed by post hoc Tukey’s test for multiple comparison. Power analysis was additionally used for estimating the sample size in this study (See Supplementary File 1).

**Table 2:** Mapping results of *C. militaris* TBRC6039 transcriptome.

Summary	Light-Programming		Dark		Average
	Glucose	Sucrose	Glucose	Sucrose	
Total clean reads (Mb)	48.81	48.79	45.15 *	44.87 *	46.91 ± 1.90
Total mapped reads to genome	39,885,625 (81.72%)	39,088,706 (80.12%)	38,097,583 (84.38%)	37,711,698 (84.05%)	38,695,903 ± 850,937.56 (82.57 ± 2.02%)
Number of expressed genes	8504	8499	8570	8542	8529 ± 33.54
All expressed genes	8747				

* Data were taken from the previous work [17].

**Table 3:** List of reporter metabolites of *C. militaris* at the light-programming condition.

Reporter Metabolites	Up-Directional *p*-Value
AMP	0.0020 *
ADP	0.0020 *
ATP	0.0020 *
UDP	0.0020 *
glucose	0.0020 *
UDP-glucose	0.0020 *
thioredoxin dithiol	0.0200
2-oxoglutarate	0.0798

Note: The metabolite with a distinct up-directional *p*-value < 0.01 was identified as a significant reporter metabolite (*).

## Supplementary Materials

The following are available online at https://www.mdpi.com/2079-7737/9/9/242/s1, Supplementary File 1: Phenotypic characteristics obtained from the glucose and sucrose cultures of *C. militaris* at light-programming and dark conditions, Supplementary File 2: Mapping results of *C. militaris* TBRC6039 transcriptome, Supplementary File 3: Functional annotation of expressed genes of *C. militaris*, Supplementary File 4: List of differentially expressed genes (DEGs) between light-programming and dark conditions of the glucose or sucrose cultures, Supplementary File 5: The improved genome-scale metabolic network (GSMN) of *C. militaris*.
